# Myeloid Translocation Gene-16 Co-Repressor Promotes Degradation of Hypoxia-Inducible Factor 1

**DOI:** 10.1371/journal.pone.0123725

**Published:** 2015-05-14

**Authors:** Parveen Kumar, Urban Gullberg, Inge Olsson, Ram Ajore

**Affiliations:** Department of Hematology, Lund University, Lund, Sweden; University of Dundee, UNITED KINGDOM

## Abstract

The myeloid translocation gene 16 (MTG16) co-repressor down regulates expression of multiple glycolytic genes, which are targets of the hypoxia-inducible factor 1 (HIF1) heterodimer transcription factor that is composed of oxygen-regulated labile HIF1α and stable HIF1β subunits. For this reason, we investigated whether MTG16 might regulate HIF1 negatively contributing to inhibition of glycolysis and stimulation of mitochondrial respiration. A doxycycline Tet-On system was used to control levels of MTG16 in B-lymphoblastic Raji cells. Results from co-association studies revealed MTG16 to interact with HIF1α. The co-association required intact N-terminal MTG16 residues including Nervy Homology Region 1 (NHR1). Furthermore, electrophoretic mobility shift assays demonstrated an association of MTG16 with hypoxia response elements (HREs) in *PFKFB3*, *PFKFB4* and *PDK1* promoters *in-vitro*. Results from chromatin immunoprecipitation assays revealed co-occupancy of these and other glycolytic gene promoters by HIF1α, HIF1β and MTG16 in agreement with possible involvement of these proteins in regulation of glycolytic target genes. In addition, MTG16 interacted with prolyl hydroxylase D2 and promoted ubiquitination and proteasomal degradation of HIF1α. Our findings broaden the area of MTG co-repressor functions and reveal MTG16 to be part of a protein complex that controls the levels of HIF1α.

## Introduction

Co-repressors modulate gene expression by controlling activities of transcription factor complexes. For example, restoration/elevation of the myeloid translocation gene 16 (**MTG16**) co-repressor inhibited expression of key genes of glucose metabolism, which diminished glycolysis, stimulated mitochondrial respiration and decreased cell cycle activity [1]. The conserved MTG co-repressor gene family with similarity to *Nervy* in Drosophila also accommodates ***MTG8*** (or eight-twenty one, *ETO*) and MTG-related protein-1 (***MTGR1***) [2]. MTG16 is the most highly expressed isoform in hematopoietic stem/progenitor, erythroid, megakaryocytic, and B cells [3,4]. Notably, all MTG genes are targets of chromosomal translocations in leukemia as fusion partners to the gene encoding the transcription factor AML1 [5–10]. Apparently, MTG co-repressors bind only indirectly to DNA, by interactions with transcription factors. The Nervy Homology Regions (NHRs) of MTG proteins are responsible for additional interactions with co-repressor proteins such as Swi-independent 3A (SIN3A), nuclear receptor co-repressor 1 (N-CoR) and silencing mediator for retinoid and thyroid receptors (SMRT) [11,12]. The multi—protein complexes formed recruit histone deacetylases (HDACs) [13], which catalyze gene repression by lysine deacetylation of histones [14–17] with an essential epigenetic role in gene transcription.

Mature cells normally cover energy needs for homeostasis by ATP generated through mitochondrial respiration. In contrast, neoplastic cells characteristically show low mitochondrial respiration paralleled with increased glucose uptake, lactate export and extracellular acidification, even under good oxygen supply, termed the Warburg effect [18]. This glycolytic switch driven by activation of oncogenes and inactivation of suppressor genes [19,20] supports anabolic needs for metabolic intermediates required for neoplastic cell growth [20]. Highly proliferating normal cells also show stimulated glycolysis, which is driven by proliferative signals from growth factors [21]. We discovered that the MTG16 co-repressor inhibited the glycolytic switch and stimulated mitochondrial respiration [1]. In support of this, expression of hypoxia—inducible transcription factor 1 (HIF1) glycolytic target genes such as 6-phosphofructo-2-kinase/fructose-2,6-biphosphatase 3 (PFKFB3) [22,23] and PFKFB4 [24] as well as the mitochondrial respiration inhibitor pyruvate dehydrogenase kinase isoenzyme 1 (PDK1) [25] were inhibited. The inhibition may involve negative regulation of HIF1 by MTG16.

HIF1 is a heterodimer composed of oxygen—regulated unstable HIF1α and ubiquitously expressed stable HIF1β subunits [26]. At hypoxic conditions, the oxygen—dependent proteasomal degradation of HIF1α is inhibited [27] permitting dimerization with HIF1α, binding of dimer to hypoxia response elements (HREs) and transcriptional activation of target genes [28,29]. HIF1 controls cellular adaptation to oxygen lack [30,31] by activating genes that coordinate stimulated glycolysis with repressed mitochondrial respiration [32]. However, HIF1α may stay active in neoplastic cells even at non—hypoxic oxygen concentrations being activated by molecules such as pyruvate, lactate, mammalian target of rapamycin (mTOR), reactive oxygen species (ROS), and oncogene gain of function or suppressor gene loss of function as reviewed in [33].

Given our findings of MTG16–mediated inhibition of glycolysis and stimulation of mitochondrial respiration, we asked whether the MTG16 co-repressor might regulate HIF1 negatively and contribute to the inhibited expression of glycolytic genes such as *PFKFB3*, *PFKFB4* and *PDK1* that are down regulated when MTG16 is elevated. For support, we first determined whether MTG16 is a HIF1α-interacting protein. Then, we investigated whether MTG16 is part of a HIF1–containing protein complex at target promoters. Furthermore, we investigated whether ectopically expressed MTG16 affected HIF1α stability. Controlled biosynthesis of ectopically expressed MTG16 was obtained by use of a doxycycline—regulated Tet-On time and dose—dependent gene expression system in B-lymphoblastic Raji cells [1].

## Materials and Methods

### Cell Culture

The Burkitt's lymphoma human Raji cells [34] were grown in RPMI-1640 medium containing 10% Fetal Bovine Serum (FBS) (Gibco BRL, Life Technologies, Rockville, MD) and supplemented with 11.1 mM glucose. Monkey kidney COS-7 cells [35] were grown in DMEM medium containing 10% FBS. All cell lines were from ATCC.

### Transfection

Raji cells (8x10^6^) were electroporated with plasmid in 0.4 ml of culture medium using the Bio-Rad Electroporation Apparatus (Bio-Rad Laboratories, Hercules, CA) with electrical settings of 960 mF and 260V. Antibiotic was added after 48 h for selection of resistant recombinant clones, which were isolated, expanded into mass cultures and screened for expression.

### Generation of stable doxycycline inducible *MTG16* clones

The Tet-On 3G tetracycline inducible gene expression system (Clontech, Ozyme, Saint Quentin en Yulines, France) was used for generation of stable doxycycline inducible clones of *MTG16* inserted under the TRE3G promoter (P_TRE3G_) in B-lymphoblastic Raji cells (Raji/MTG16 Tet-On 3G cells) as previously described [1]. Incubation with 10–20 ng/ml of the tetracycline analog doxycycline induces Tet-On 3 G trans activator binding to tet operator repeats within P_TRE3G_ leading to transcriptional *MTG16* activation. MTG16 biosynthesis was seen after 3 to 4 h of induction at a very low concentration (20 ng/ml) of doxycycline making unspecific effects unlikely (data not shown).

### Quantitative real time polymerase chain reaction (qPCR)

RNA was isolated using RNAeasy mini kit # 74104 (Qiagen, Valencia, CA). After isolation, RNA was incubated with DNase I, #EN0521 (Fermentas Inc, Glen Burnie, MD) for 30 min at 37°C. Then cDNA was synthesized using omniscript RT kit #20511 (Qiagen, Valencia, CA). The QPCR reaction contained 7.5 μl 2x MAXIMA SYBR mix (Fermentas Inc, Glen Burnie, MD), 0.5 μmoles (0.5 μl) of each primer, 2 μl cDNA template and water to a final volume of 15 μl. PCR parameters were: 50°C for 2 min, 95°C for 10 min, 40 × (95°C for 15 sec, 60°C for 30 sec and 72°C for 30 sec). Primers were designed as shown (S1 Table). Human *18S rRNA* and *GAPDH* were used as references. Relative quantification values were expressed using the ΔΔCt method normalized to the reference genes and related to the expression of the controls [36]. Normalization: ΔCt = Ct (sample)—Ct (geomean of Ct of GAPDH and 18S rRNA). ΔΔCt = ΔCt (sample)- ΔCt (control). Relative quantification = 2 –^**ΔΔCt**^

### Chromatin Immunoprecipitation (ChIP) assays

ChIP was performed as described previously [37]. For IP, 2 μl polyclonal anti-MTG [38], mouse polyclonal anti-HIF-1a (#ab2185, Abcam, Cambridge, UK), mouse monoclonal anti-HIF-1b (#ab2, Abcam, Cambridge, UK), mouse polyclonal anti-b-actin (# sc8432), (SantaCruz, CA) were used. List of primers used for real time PCR amplification of HRE regions of PDK1, PFKFB3, PFKFB4, HK, PFK, LDHA and control regions are mentioned in S1 Table. qPCR was performed with 2 ml of each CHIP DNA sample in duplicate using SybrGreen (MAXIMA SYBR mix, Fermentas Inc, Glen Burnie, MD) and the ABI StepOnePlus real time PCR system and normalized to input. Identical amounts of input-DNA were used for IP with specific antibody or control β-actin antibody. Fold enrichment was calculated based on Ct as 2^ΔΔCt^, where ΔCt = Ct_IP_—Ct_input_ and ΔΔCt = ΔCt_antibody_- ΔCt_β-actin_ [39].

### Co-immunoprecipitation

Co-immunoprecipitation was performed as described earlier [40]. Raji-MTG16 cells were incubated overnight under 4% O_2_ with doxycycline for expression of MTG16. Alternatively, COS-7 cells were transfected with MTG16 and HIF1α and harvested after 24 h. The cells were homogenized with lysis buffer (250 mM NaCl, 20 mM Na-phosphate /pH 7.0/, 30 mM Na-pyrophosphate /pH 7.0/, 5 mM EDTA, 0.1 mM Na_3_VO_4_, 10 mM NaF and 0.1%NP-40) supplemented with protease inhibitors on ice for 1h. Lysates were cleared by centrifugation at 13000 rpm for 1 h at 4°C and incubated overnight with Protein A Sepharose and antibody. The Protein-A-Sepharose beads were then washed three times in lysis buffer to remove unbound non-specific protein. Thirty-μl sample buffer (114 mM Tris HCL /pH 6.8/, 15% glycerol, 105 mM SDS, 238 mM β-mercaptoethanol and 0.013% Bromophenol blue) was added followed by boiling 5 min, centrifugation and Western blotting.

### Immunoblot assays

Western blotting was performed essentially as previously described [4], using the following antibodies: Polyclonal α-MTG reactive with all MTG homologues [38]; rabbit polyclonal anti-histone H3 CHIP grade (# ab1791), rabbit polyclonal α-HIF1α (# ab2185), mouse monoclonal α-HIF1β (# ab2) (Abcam, Cambridge, UK); mouse monoclonal anti-actin (#sc8432) (Santa Cruz, CA); rabbit polyclonal α-PHD2/HIF prolyl hydroxylase 2 antibody (# NB100-137) (Novus Biologicals, Littleton, CO); rabbit monoclonal α-hydroxy-HIF1α (Pro-564) (#D43B5) (Cell Signalling, Danvers, MA); mouse monoclonal α-FLAG antibody (# F3165), (Sigma, St. Louis, MO).

### GST pulldown assays

GST and GST fusion proteins were expressed in Escherichia coli JM109 cells by transformation with GST, GST-HIF1α and GST-MTG16 in pGEX-4T3 vectors. Recombinant bacterial cells expressing GST, GST-HIF1α and GST-MTG16 were lysed in NTN buffer (100 mM NaCl, 20 mM Tris and 0.1%NP-40) by sonication for 10 min. Lysates were cleared by centrifugation 10 min at 13000 rpm. GST-HIF1α and GST-MTG16 were bound to glutathione-Sepharose beads during rotation 2 h at 4°C. The beads were washed five times in NTN buffer containing 1.0 mM EDTA. Then, whole cell lysate was mixed with beads and incubated during rotation overnight at 4°C. After washing five times with NTN buffer containing 1.0 mM EDTA, bound proteins were eluted with sample buffer during heating at 95°C for 5 min and examined by Western blotting.

### Ubiquitination Assay

Raji-MTG16 cells were transfected with pcDNA3-FLAG-UBIQ and pcDNA3-HA-HIF1α and incubated in 4% oxygen for 24 h. Cells were harvested, lysed and co-immunoprecipitated with mouse monoclonal anti-HA antibody (# sc7392), (Santa Cruz, CA) overnight and examined by Western blotting.

### Statistical analysis

The significance of difference between samples was determined by the unpaired Student´s *t* tests or the one- or two-way ANOVA followed by *post hoc* tests using the Graphpad Prism version 5.0a Software (GraphPad Software, Inc., CA), unless stated differently. Single, double and triple asterisks represent *P<0*.*05*, *P<0*.*001* and *P < 0*.*0001*, respectively. Data are presented as means±SEM.

## Results

### MTG16 is a HIF1α–interacting protein

**Fig 1:**
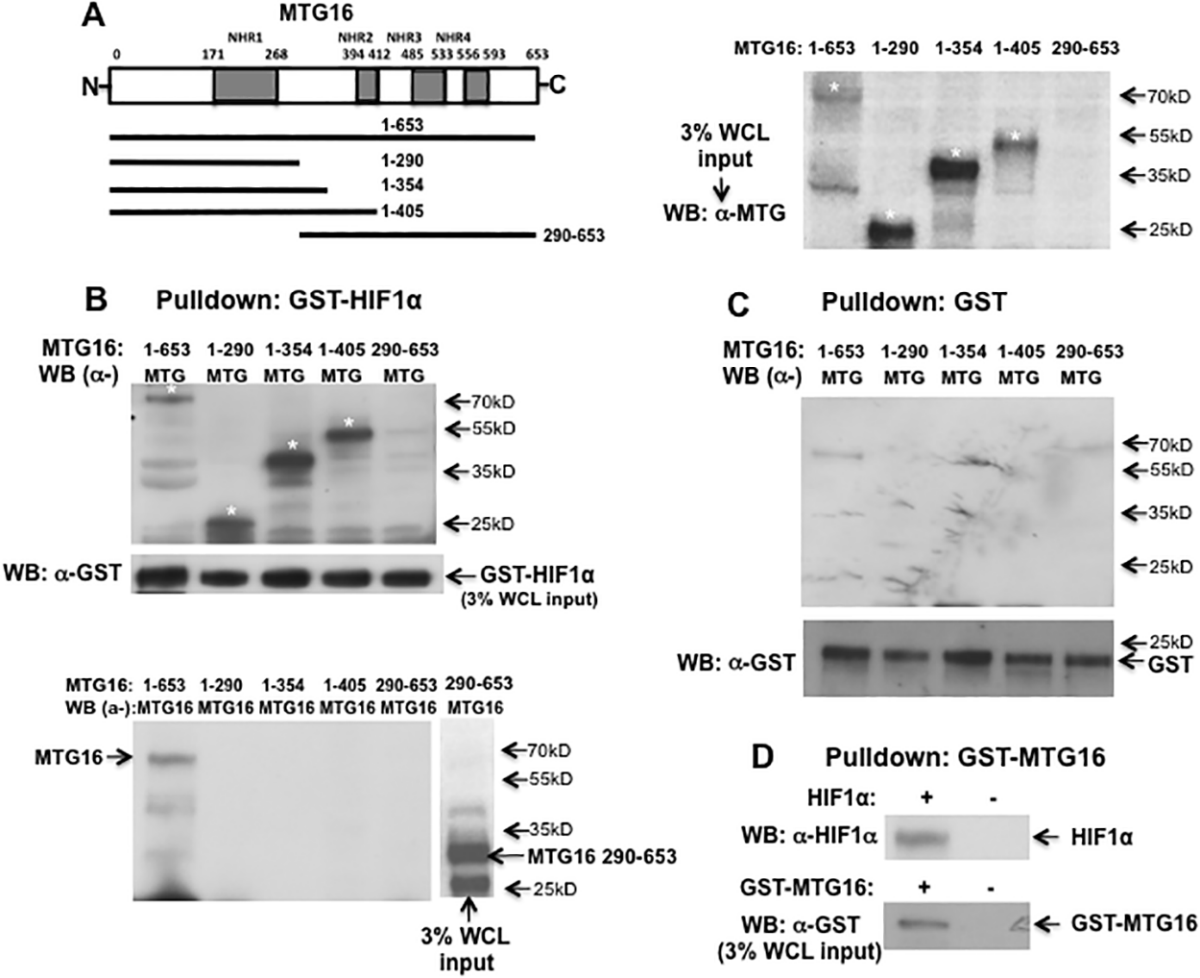
MTG16 binds to HIF1a in-vitro. **A.** Scheme of MTG16 protein with Nervy Homology Regions (NHRs) marked. Truncation constructs investigated are also shown. **B**. GST pulldown assays using GST-HIF1α were performed on whole cell lysates (WCL) of COS-7 cells expressing MTG16 or the MTG16 truncation constructs indicated in panel A. For detection of pulled down MTG16 constructs by Western blotting (WB) two antibodies were used; α-MTG was raised against amino acids 31–250 of MTG8 and recognizes wild type and truncation constructs of MTG16 with intact NHR1 [41]. α-MTG16 was raised against amino acids 452–466 of MTG16 [38]. The MTG16 constructs pulled down by GST-HIF1α and detected by α-MTG (upper panel) are marked with stars. No signal was detected for the 290–653 construct (lane 5) as it lacks the epitope for α-MTG antibody. Using the same blotting membrane as in upper panel, the middle panel shows detection by α-MTG16 of full length MTG16 (lane 1) but not MTG16 290–653 construct (lane 5) demonstrating lack of pulldown of the latter construct. Serving as a positive control, α-MTG16 detected the MTG16 290–653 construct in WCL (lane 6). The third panel shows input in whole cell lysate (WCL) of the MTG16 constructs detected by α-MTG and marked with stars. **C**. To rule out unspecific binding to GST, pull-down assays using GST were performed on WCL of COS-7 cells expressing MTG16 or the MTG16 truncation constructs indicated. No pull-down was observed indicating lack of unspecific binding to GST. The WCL input of GST is shown at bottom. **D**. GST pull-down assays using GST-MTG16 were performed on WCL of COS-7 cells transfected with HIF1α. Pull-down of HIF1α is shown (upper lane). The lower lane shows the WCL input of GST-MTG16. One out of at least three experiments is shown in B to D.

In order to detect a direct interaction between HIF1α and MTG16 and to map possible interaction domains, we used bacterially produced fusions of HIF1α with glutathione-S-transferase (GST). GST-HIF1α was recovered on glutathione—Sepharose beads and examined by SDS-PAGE. The binding of MTG16 species to HIF1α in cell lysates from COS-7 cells transfected with MTG16 was investigated. Results showed full length MTG16 to be pulled down by GST-HIF1α (Fig 1B). A number of MTG16 truncation constructs were used to map sites of interaction with HIF1α. MTG16 residues 1–290, 1–354, 1–405 bound to GST-HIF1α (Fig 1B). However, a construct consisting of residues 290 to 653 (lacking the NHR1 domain) was not pulled down by GST-HIF1α. As follows, MTG16 residues 1–290 containing NHR1 (residues 171–268) are sufficient for binding to HIF1α. Binding specificity was confirmed by lack of interaction of MTG16 with GST (Fig 1C). Furthermore, in the reverse experiment, full length HIF1α was pulled down by GST-MTG16 (Fig 1D).

**Fig 2:**
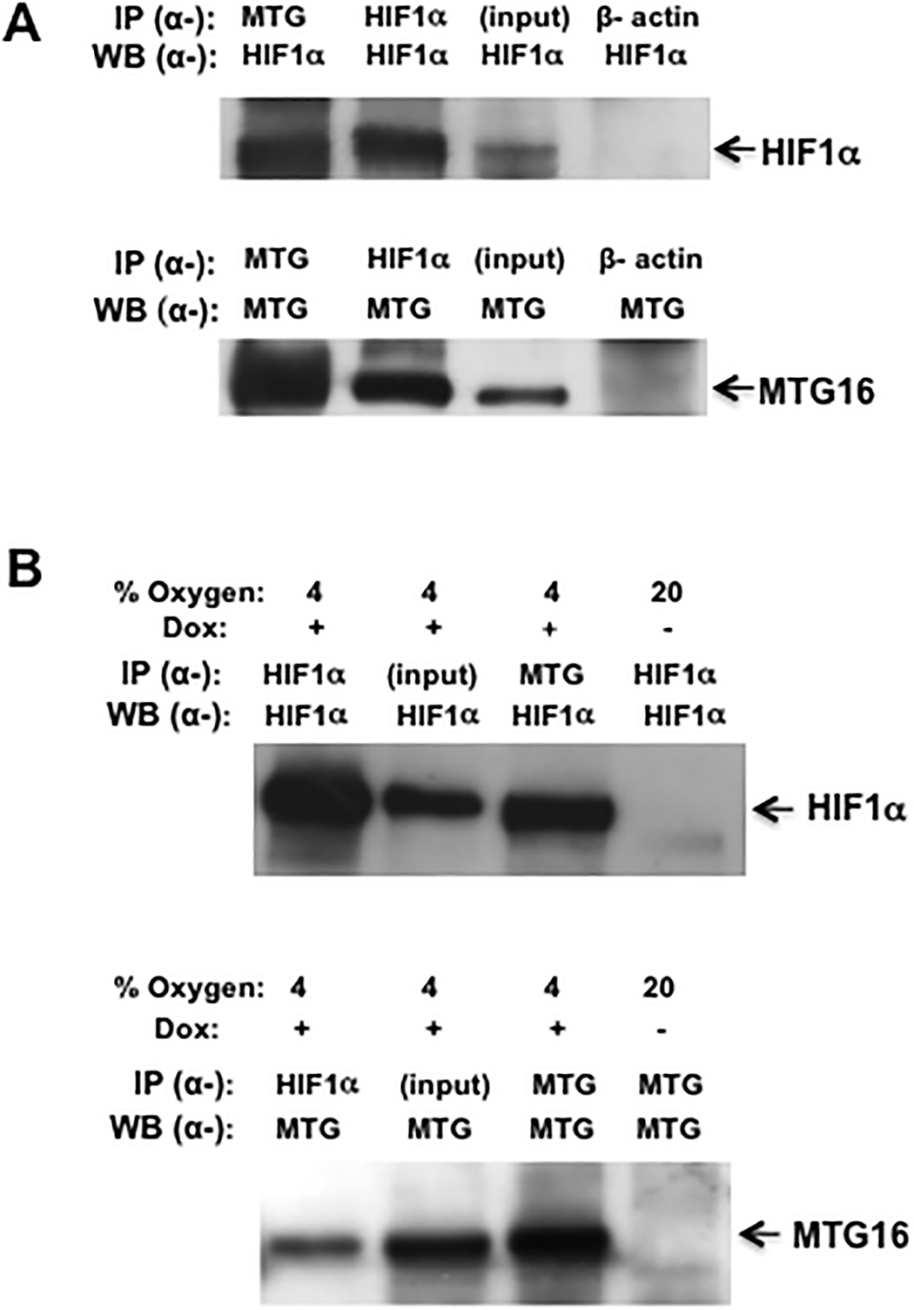
MTG16 and HIF1a co-precipitate. **A.** COS-7 cells were transfected with *MTG16* and *HIF1α* and examined by IP-Western as described in Materials and Methods using α-MTG and α-HIF1α antibodies. MTG16 (precipitated with *a*-MTG) co-precipitated HIF1α (top panel) and the reciprocal experiment showed HIF1α to co-precipitate MTG16 (lower panel). The input of HIF1α in 3% whole cell lysate and the corresponding input of MTG16 are shown in upper and lower panel, respectively. Control experiments with α-β-actin showed lack of non-specific co-precipitation of HIF1α or MTG16 (lanes 4). The same membrane was used for the immunoblotting in upper and lower panel. **B**. Similar experiments as in A were carried out on Raji-MTG16 cells, which were induced for 12 h with 20 ng/ml doxycycline (dox) to express MTG16 and exposed to 4% O_2_ to induce HIF1α. MTG16 (precipitated with α-MTG) co-precipitated HIF1α (top panel) and the reciprocal experiment showed HIF1α to co-precipitate MTG16 (lower panel). The input of HIF1α or MTG16 in 3% of whole cell lysate is shown in lanes 2. As negative control, cells under exposure to 20% O_2_ not incubated with doxycycline showed no reaction with α-HIF1α or α-MTG (lanes 4). One out of at least three experiments performed is shown in A and B.

To detect interaction between MTG16 and HIF1α co-immunoprecipitation assays were used. Ectopic interactions were examined in COS-7 cells transiently transfected with *MTG16* and *HIF1α*. Results from IP-Western analyses showed that MTG16 co-precipitated HIF1α and the reciprocal IP-Western experiment showed HIF1α to co-precipitate MTG16 (Fig 2A).

Interaction between HIF1α and MTG16 was also examined in Raji/MTG16 Tet-On 3G cells, which were incubated with doxycycline to induce expression of MTG16. The experiments were performed during exposure to 4% O_2_ to increase the level of HIF1α. Results from IP-Western analyses showed that MTG16 co-precipitated HIF1α (Fig 2B, top panel) and the reciprocal experiment showed that HIF1α co-precipitated MTG16 (Fig 2B, lower panel). These data demonstrate binding between MTG16 and HIF1α.

### MTG16 is detectable at HIF1α response elements (HREs) in *PFKFB3*, *PFKFB4* and *PDK1* promoters *in-vitro*

**Fig 3:**
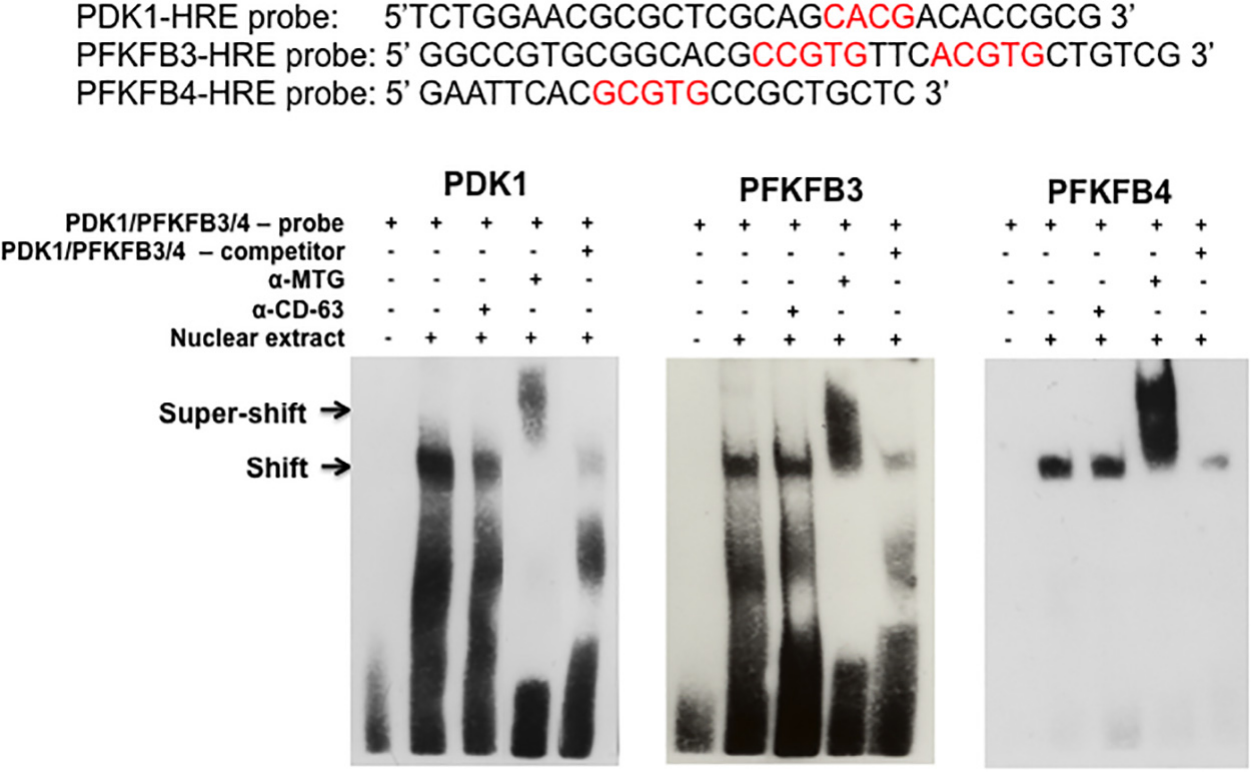
MTG16 binds to HIF1a response elements in promoter as shown by electrophoretic mobility shift/supershift assays. Sequences for oligonucleotide probes used containing the HIF1α response elements (HRE) and flanking regions of PDK1, PFKFB3 or PFKFB4 promoters are depicted. Nuclear extract was from Raji-MTG16 cells incubated with 20 ng/ml of doxycycline for 12 h to induce expression of MTG16 under exposure to 4% O_2_ to induce HIF1α. The primary DNA-nuclear protein interactions are depicted by arrows marked shift and the DNA-nuclear protein—antibody interactions are depicted by arrows marked supershift. A shift is shown for all probes that is competed for by unlabeled probe indicating specific binding of nuclear extract protein to the biotinylated probe containing the HRE. Protein bound both to PDK1, PFKFB3 and PFKFB4 probes was "super—shifted" by antibody to MTG16 (α-MTG) but not with antibody to CD63 (α-CD63) indicating binding of MTG16 to the probe. The results suggest that MTG16 may bind to HIF1α at the PDK1, PFKFB3 and PFKFB4 promoters. One out of three experiments performed is shown.

As MTG16 does not bind directly to DNA, indirect association might be mediated via a protein complex containing HIF1α. Electrophoretic mobility shift/supershift assays (EMSA) were used to examine the capacity for association with HREs in *PFKFB3*, *PFKFB4* and *PDK1* promoters. Biotinylated oligonucleotide probes of HRE core consensus sites with flanking regions (Fig 3) were incubated with nuclear extracts prepared from Raji-MTG16 cells incubated at 4% O_2_ to increase HIF1 and with doxycycline for 12 h to induce production of MTG16 in order to investigate interactions between probe and protein. As a result, nuclear extract proteins showed a shift indicating binding of protein to the probes (Fig 3). Binding was specific as no shift was observed in the presence of excess unlabeled competitor probe. Furthermore, protein bound to the labeled probes was “super—shifted” by α-MTG antibody but not with a control antibody. Our results show that MTG16 becomes associated with HREs within *PFKFB3*, *PFKFB4* and *PDK1* promoters. In addition to the specific shift is an unspecific band—shift for the PDK1 and PFKFB3 promoters, which is unaffected or only weakly affected by the competitor probe (Fig 3). Nonetheless, the band contains MTG16 as it is lost by super-shifting with α-MTG antibody. In conclusion, EMSA demonstrated a capacity of MTG16 to associate with HREs in *PFKFB3*, *PFKFB4* and *PDK1* promoters *in-vitro*.

### MTG16, HIF1α and HIF1β co-occupy hypoxia response elements (HREs) in *PFKFB3*, *PFKFB4* and *PDK1* promoters *in-vivo*.

**Fig 4:**
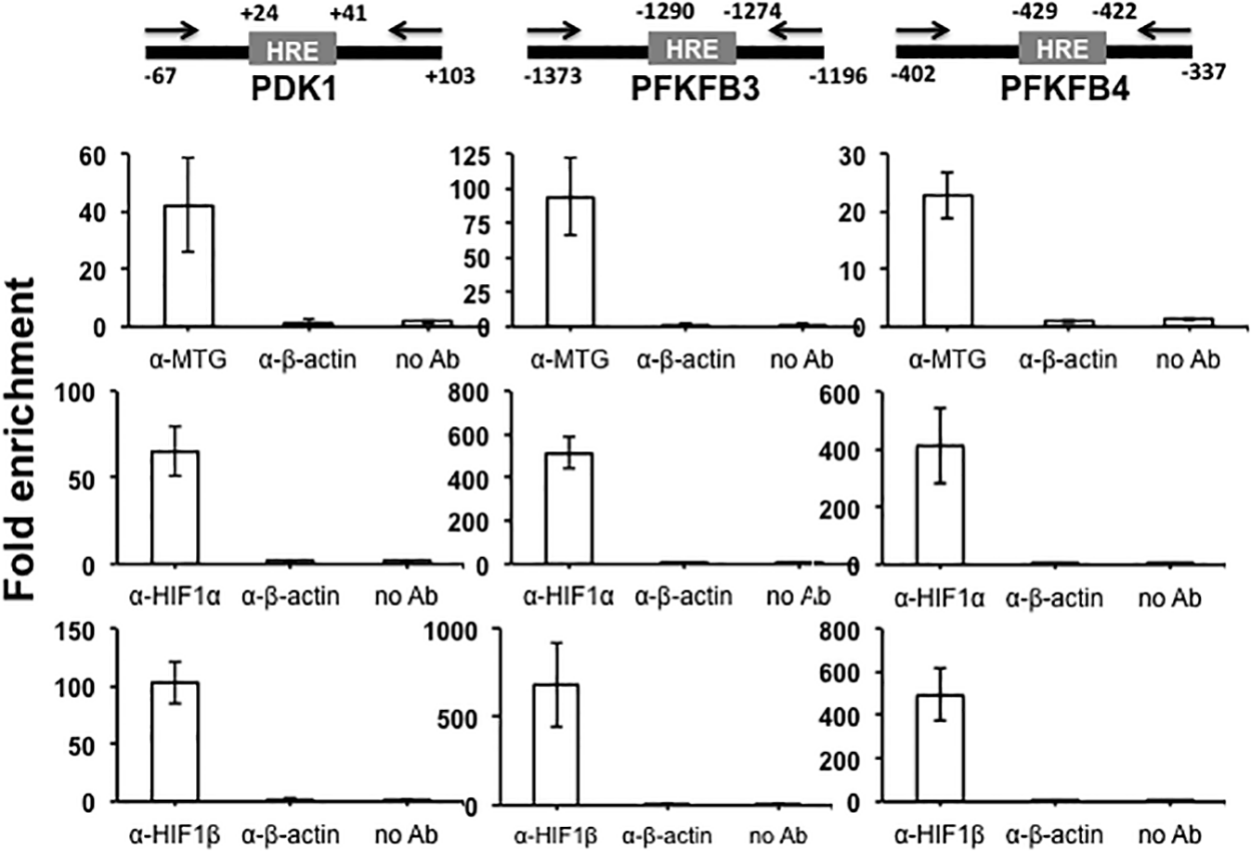
MTG binds to HIF1a response elements in-vivo in hypoxia as shown by chromatin immunoprecipitation (ChIP). ChIP assays were carried out using chromatin isolated from Raji-MTG16 cells incubated at **4% O**_**2**_ and induced with 20 ng/ml of doxycycline 12 h to express MTG16. Schematics of promoter regions of PDK1, PFKFB3 or PFKFB4 including HRE are shown. Numbers are relative to transcription start site (TSS). Positions of primers for real time PCR are indicated. The numbers indicate nucleotide position in the promoters corresponding to TSS. The protein-DNA complexes were immunoprecipitated with control α-β-actin, α-MTG (detecting MTG16), α-HIF1α or α-HIF1β antibodies and analyzed by qPCR (mean ± SEM, n = 3). Enrichment of MTG16, HIF1α and HIF1β on HRE is shown. No enrichment was seen with control antibody or without antibody.

**Fig 5:**
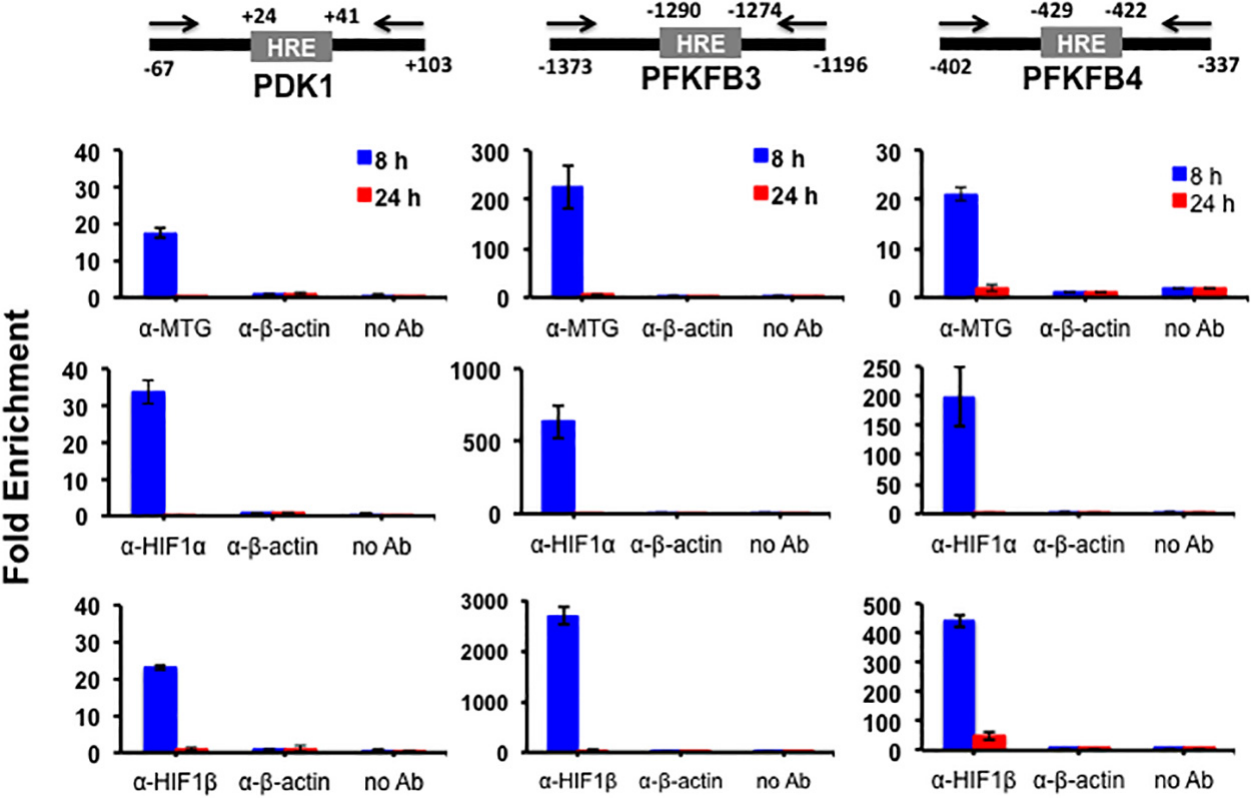
MTG binds to HIF1a response elements in-vivo in normoxia as shown by chromatin immunoprecipitation (ChIP). ChIP assays were carried out as described in Fig 4 but chromatin was isolated from Raji-MTG16 cells incubated under **20% O**_**2**_ with 20 ng/ml of doxycycline for 8 or 24 h to induce expression of MTG16. Using chromatin extracted from the cells enrichment of MTG16, HIF1α and HIF1β on HRE is shown at 8 h of doxycyline incubation whereas no enrichment is observed at 24 h of incubation (mean ± SEM, n = 3). No enrichment was seen with control antibody or without antibody. Results from three experiments performed are shown.

To get further support for an interaction between MTG16 and HIF1α on promoters, ChIP assays were used. We asked whether MTG16 was present in HIF1–containing complexes. Thus, ChIP assays were performed to detect MTG16, HIF1α and HIF1β co-occupancy *in-vivo* at HREs in the promoters of the HIF1 target genes. The assays were performed with chromatin isolated from Raji/MTG16 Tet-On 3G cells incubated at 4% O_2_ (to activate HIF1α) and with doxycycline 12 h (to induce expression of MTG16) after which immunoprecipitation with antibodies towards MTG16, HIF1α or HIF1β was performed. By the use of primers specific for HRE sites with flanking regions, qPCR showed amplification products generated by precipitation with either α-MTG, α-HIF1α or α-HIF1β, indicating binding *in-vivo* of MTG16, HIF1α and HIF1β near HRE sites of *PFKFB3*, *PFKFB4* and *PDK1* (Figs 4 and 5).

ChIP assays were also carried out with chromatin isolated from Raji/MTG16 Tet-On 3G cells incubated at 20% O_2_ (Fig 5) and with doxycycline for 8 or 24 h to induce expression of MTG16. The results indicated binding *in-vivo* of MTG16, HIF1α and HIF1β near HRE sites of *PFKFB3*, *PFKFB4* and *PDK1* after 8 h but not after 24 h of incubation with doxycycline (Fig 5). The lack of MTG16 binding of HIF1α and HIF1β at 24 h is consistent with the MTG16–mediated total downregulation of HIF1α that was observed at this time point in cells exposed to 20% O_2_ (shown below) and serves as an additional control for specificity of MTG16 binding to HIF1α. In as much as the HIF1α expression was much lower at 20% O_2_ compared to 4% O_2_, the results of Fig 5 may be less biologically relevant. However, down regulation of *PDK1*, *PFKFB3* and *PFKFB4* was observed previously during the conditions of 20% O_2_ [34] consistent with a correlation between binding of the MTG16-HIF complex and transcriptional repression.

**Fig 6:**
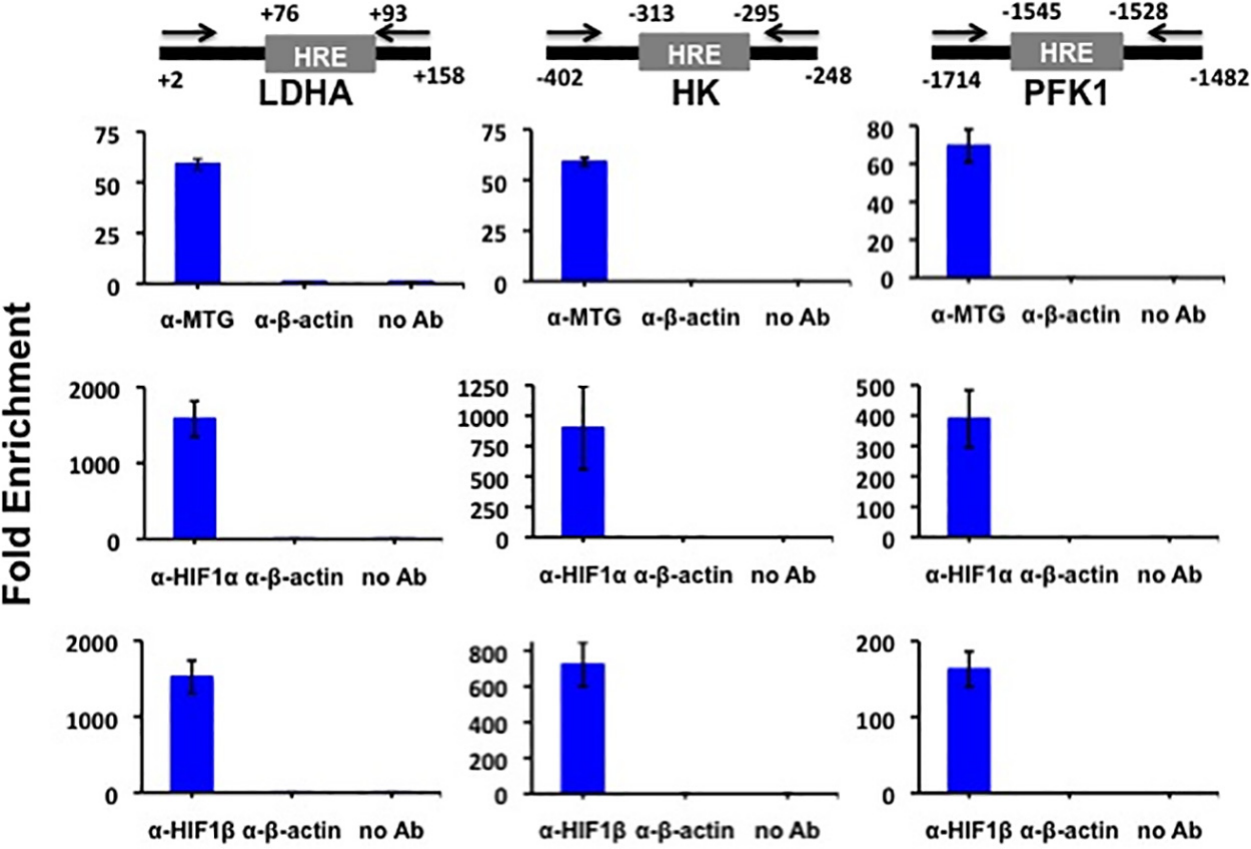
Chromatin immunoprecipitation (ChIP) analysis is consistent with in-vivo interaction of MTG16 and HIF1a on the promoters of LDH, HK and PFK. ChIP assays were carried out as described in Materials and Methods using chromatin isolated from Raji-MTG16 cells incubated under exposure to 4% O_2_ with 20 ng/ml of doxycycline for 8 h to induce production of MTG16. Schematic drawings of promoter regions including HRE are shown. Numbers are relative to TSS. Positions of primers for real time PCR are indicated. The protein-DNA complexes were immunoprecipitated with α-MTG (detecting MTG16), α-HIF1α, α-HIF1β or α-β-actin antibodies and analyzed by qPCR (mean±SEM, n = 3). Enrichment of MTG16, HIF1α and HIF1β on HRE is shown. No enrichment was seen without antibody or with α-β-actin antibody.

HIF1 activates many glycolytic target genes in addition to *PFKFB3*, *PFKFB4* and *PDK1* such as lactate dehydrogenase A (*LDHA*), hexokinase (*HK*) and phosphofructokinase-1 (*PFK1*) [41]. The promoters of these genes were also strongly downregulated by MTG16 (S1 Fig). Therefore, we determined whether MTG16 also interacted *in-vivo* with bound HIF1α on the *LDH*, *HK* and *PFK1* promoters. ChIP assays showed binding of MTG16, HIF1α and HIF1β near HRE sites also at these promoters (Fig 6). The endogenous co-occupancy of glycolytic gene promoters by both HIF1α and MTG16 is consistent with the interaction that we detected between these two proteins.

### MTG16 promotes HIF-1α degradation

**Fig 7:**
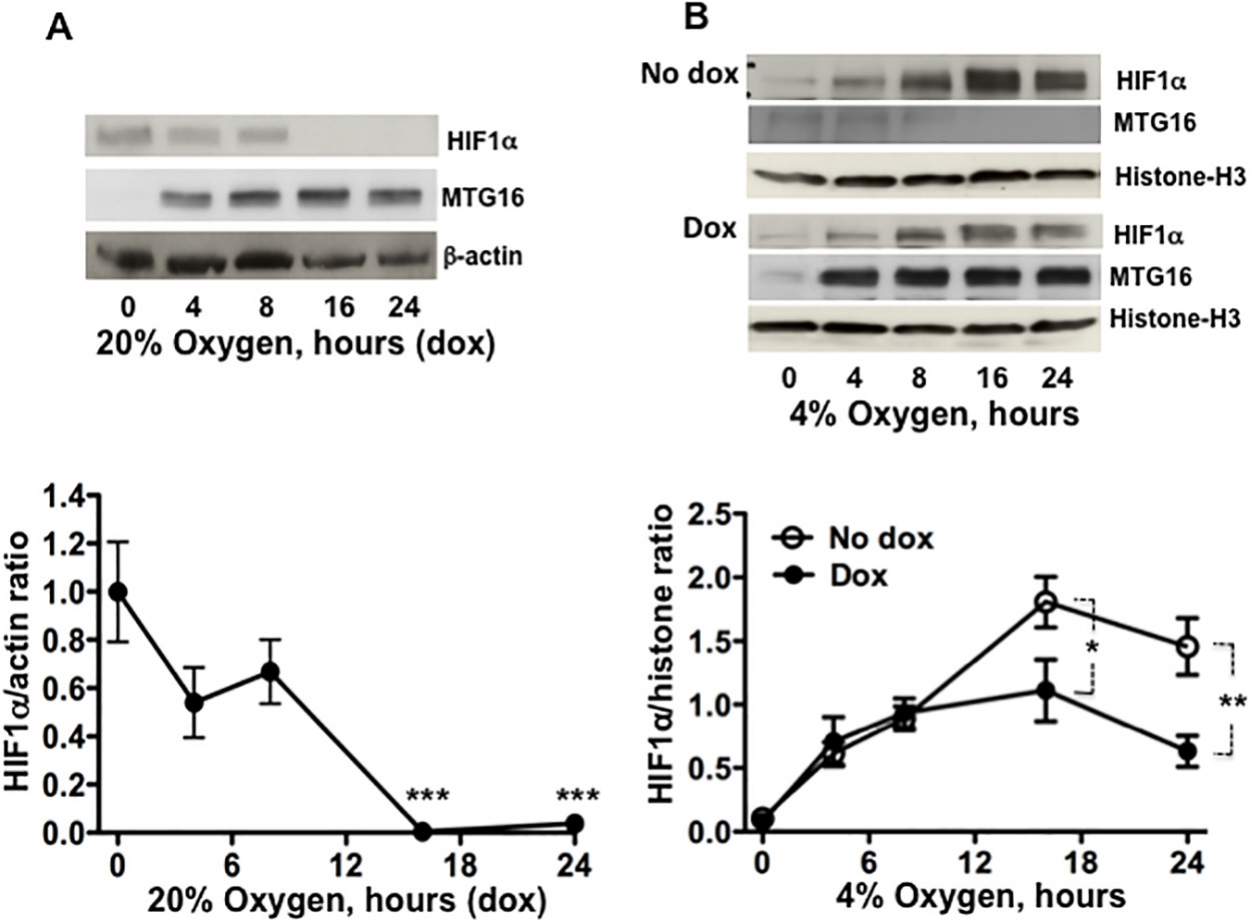
MTG16 reduces the level of HIF1a. **A**. The level of HIF1α was examined by Western blotting in lysates from Raji-MTG16 cells incubated under exposure to 20% O_2_ with 20 ng/ml of doxycycline to induce synthesis of MTG16 (as detected by α-MTG). HIF1α becomes undetectable after 8 to 16 h of incubation. Blotting data from one out of three experiments is shown (top). The intensity of the bands was quantified by densitometry and expressed as HIF1α/β-actin ratio, n = 3. The level of HIF1α was reduced after 16 h; n = 3, ***P<0.001(bottom). **B**. The level of HIF1α was examined by Western blotting using the α-MTG antibody in lysates from Raji-MTG16 cells incubated under hypoxia with 4% oxygen with or without 20 ng/ml of doxycycline to induce synthesis of MTG16. Blotting data from one out of three experiments is shown (top). To compensate for a lower level of HIF1α at normoxia compared to hypoxia, the exposure time of photographic film was 60 sec for experiments performed at 20% O_2_ (A) compared to 20 sec for experiments carried out at 4% O_2_ (B). The intensity of the bands was quantified by densitometry and expressed as HIF1α/β-actin ratio. The level of HIF1α was reduced after 24 h; n = 3, **P<0.01(bottom).

**Fig 8:**
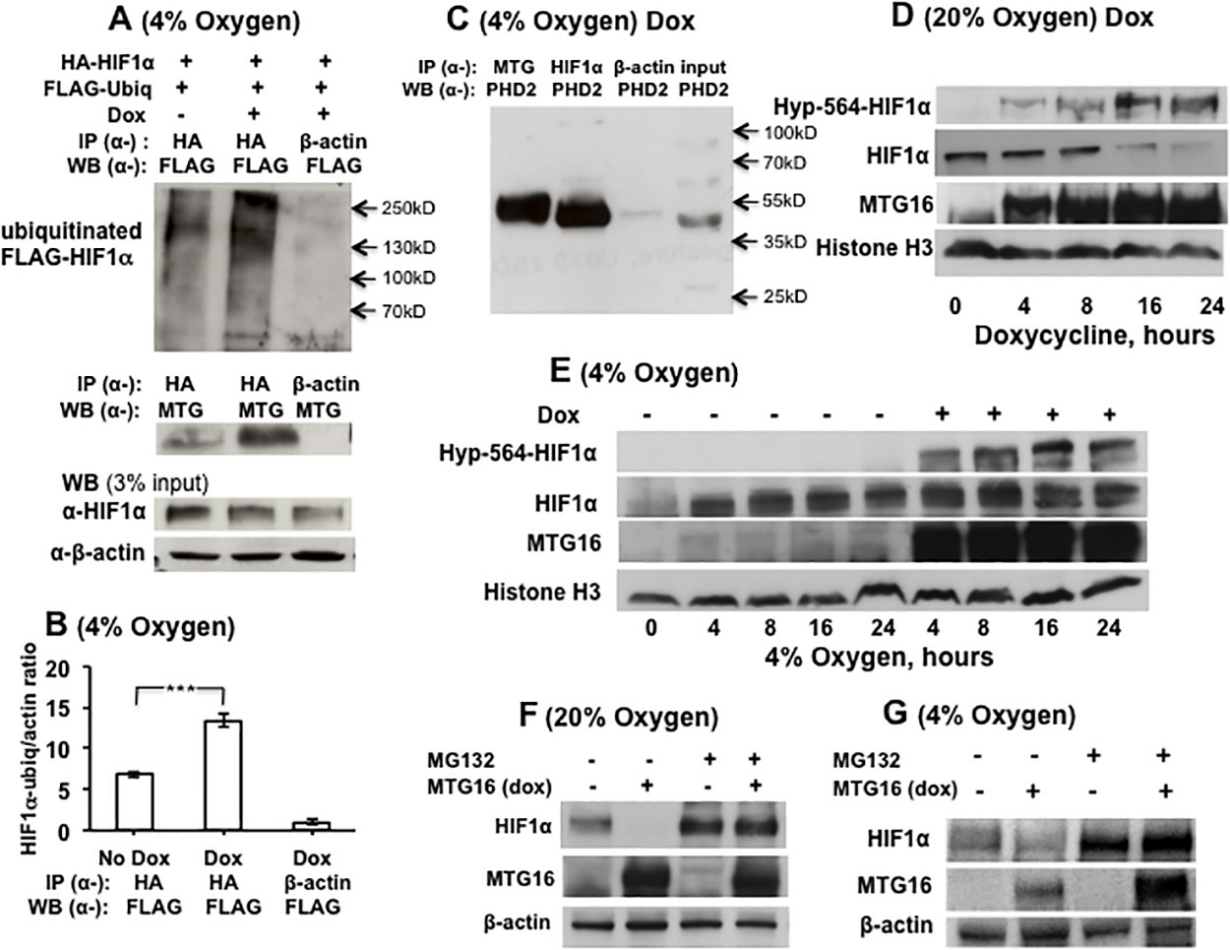
MTG16 interacts with PHD2 and induces ubiquitination and proteasomal degradation of HIF1α. **A.** Raji-MTG16 cells were co-transfected with HA-HIF1α and FLAG-Ubiquitinin and incubated 48 h at 4% O_2_ with 20 ng/ml of doxycycline inducing production of MTG16. Cell lysate was subjected to immunoprecipitation with anti α-HA antibody followed by Western blotting using α-FLAG antibody to detect ubiquitinated HIF1α, which was increased in doxycycline induced cells. Middle panel shows co-precipitation of MTG16 by α-HA but not by α-β-actin, serving as a negative control. The lower panel shows the input of HIF1α in whole cell lysate. **B.** The intensity of the HIF1α-ubiquitin bands in A (top) were quantified by densitometry and expressed as HIF1α-ubiquitin/β-actin ratio. The level of ubiquitinated HIF1α was increased in cells incubated with doxycycline; n = 3, ***P<0.001. **C**. Cells were incubated 12 h at 4% O_2_ with 20 ng/ml of doxycycline to induce production of MTG16. Both MTG16 and HIF1α co-precipitated with prolylhydroxylase D2 (PHD2) in whole cell lysates. **D.** By use of Western blotting with an anti-hydroxyproline antibody to hydroxyproline-564 in HIF1α (Hyp-564) hydroxylation of HIF1α was detected within 4 h of incubation with 20 ng/ml doxycycline at 20% O_2_. Hours on abscissa refer to beginning of incubation with doxycycline. The expected downregulation of HIF-1α with time after induction of MTG16 is seen. **E.** Experiments were performed to detect hydroxylation of HIF1α also at 4% O_2_ during incubation with 20 ng/ml doxycycline. Hours on abscissa refer to beginning of hypoxic conditions. The results confirm hydroxylation of Pro-564 in HIF1α by MTG16 at 4% O_2_. **F**. Cells incubated at 20% O_2_ with and without 20 ng/ml of doxycycline to induce production of MTG16 were co-incubated with the proteasomal inhibitor MG132 for 24 h. The inhibitor strongly protected against degradation of HIF1α. **G**. Similar experiments as in F were performed at 4% O_2_. The results confirmed strong protection against MTG16–induced degradation by MG132. The exposure of photographic film was 20 sec for A, E and G and 90 sec for D and F.

The HIF1α mRNA levels in Raji/MTG16 Tet-On 3G cells incubated with doxycycline to produce MTG16 showed no significant decrease (data not shown) indicating the co-repressor not to regulate *HIF1α* gene expression. However, the MTG16 interactions shown by IP-Western and ChIP analyses may diminish HIF1α protein stability and thereby suppress HIF1 activity. The α-MTG antibody was used in Western blotting because of its sensitivity for detection of MTGs [38]. α-MTG did not detect any MTG isoforms in Raji cells when MTG16 was not ectopically expressed (Fig 7). Therefore, this antibody detects only MTG16 in these cells when induced by incubation with doxycycline. HIF1α stability was investigated both at 20% and 4% O_2_. For detection of HIF1α by Western blotting in experiments performed at 20% O_2_, film exposures were prolonged to compensate for a lower level of HIF1α compared to conditions at 4% O_2_ as indicated in legends to Figs 7 and 8. Results from time course experiments demonstrated that MTG16 destabilized HIF1α during exposure to 20% O_2_ (Fig 7A); HIF1α became undetectable between 8 and 16 h of incubation with doxycycline. Results from similar experiments performed during exposure to 4% O_2_ (to increase the level of HIF1α) showed that HIF1α decreased after 18 to 24 h of incubation with doxycycline, as compared to incubation without doxycycline (Fig 7B). However, the decrease during hypoxia was less pronounced as compared to 20% O_2_, indicating dependency on the level of O_2_ and HIF1α.

A decrease in HIF1α protein could result from either decreased biosynthesis or increased degradation. HIF1α is hydroxylated on one of two conserved prolyl residues predominantly by prolyl hydroxylase domain—containing protein 2 (PHD2) [42] as a requisite for its polyubiquitination and proteasomal degradation. Therefore, we investigated if HIF-1α was ubiquitinated in response to MTG16. Raji-MTG16 cells co-transfected with HA-HIF1α and FLAG-ubiquitinin were incubated during exposure to 4% O_2_ with doxycycline to induce production of MTG16. Examination of immunoprecipitated HA-HIF1α by α-FLAG antibody showed that HA-HIF1α ubiquitination indeed was increased by MTG16 (Fig 8A and 8B).

Concomitant interactions of MTG16 with HIF1α and PHD2 may increase the rate of hydroxylation. We investigated binding of MTG16 to PHD2 by use of IP-Western analyses in Raji-MTG16 cells incubated during exposure to 4% O_2_ for 12 h with doxycycline to induce MTG16 production. Both MTG16 (precipitated with α-MTG) and HIF1α (precipitated with α-HIF1α) co-precipitated PHD2 in whole cell lysate (Fig 8C). This result indicates that MTG16 interacted not only with HIF1α but also with PHD2. As these co-immunoprecipitation experiments were carried out with ectopically synthesized proteins they are unlikely to reflect unspecific interactions. Further support for MTG16–mediated prolyl hydroxylation of HIF1α was achieved by immunoblotting with an antibody that specifically recognizes hydroxyproline at residue 564 of HIF1α (Hyp-564-HIF1α). Hydroxylation of Hyp-564 was detected in Raji-MTG16 cells within 4 h of incubation with doxycycline at both 20% O_2_ (Fig 8D) and 4% O_2_ (Fig 8E). The expected down regulation of HIF1α with time after induction of MTG16 is seen in both cases. The results indicate that MTG16–mediated hydroxylation may be followed by proteasomal degradation of HIF1α. In support of this, the proteasomal inhibitor MG132 strongly inhibited MTG16–mediated HIF1α decrease both during exposure to 20% (Fig 8F) and 4% O_2_ (Fig 8G).

Collectively, our results demonstrate that MTG16 reduces HIF1α protein levels through the prolyl hydroxylation—dependent pathway for ubiquitination and following proteasomal degradation.

## Discussion

Published results [1] showing that MTG16 can inhibit glycolysis and stimulate mitochondrial respiration by affecting expression of some HIF1 target genes (PFKFB3/4 and PDK1), suggested a possible negative regulation of HIF1, which controls expression of glycolytic genes in response to hypoxia / tumorigenesis. In this study, we demonstrate that the MTG16 co-repressor is an opposing regulator of HIF1 by serving as a HIF1α-interacting protein with an amplifying effect on HIF1α hydroxylation and proteasomal degradation.

HIF1 is usually not activated at non-hypoxic conditions except in certain neoplastic cells such as those used for the experiments in this work. The relevance of MTG16-mediated reduction of HIF1α observed under the non-hypoxic conditions may be questioned, as HIF1α was much lower than in the cells exposed to hypoxia. However, the complete degradation of HIF1α at 20% O_2_ concomitant with loss of MTG16 co-occupancy of glycolytic gene promoters strengthened the role of a HIF1α interaction. Total degradation of HIF1α at 20% O_2_ by MTG16 might reflect a low HIF1α level, but degradation was observed at mild hypoxia too when the HIF1α level is vigorously increased. The significance of this finding is strengthened by the reduced expression of HIF1α target genes observed in the presence of MTG16 during mild hypoxia (S1 Fig).

The MTG family of co-repressors is involved in transcriptional repression [2]. The MTG isoforms share the evolutionary highly conserved Nervy Homology Regions (NHRs) 1 to 4 [43]. The finding that the N-terminal MTG16 residues including NHR1 were sufficient for the binding of HIF1α is consistent with this domain being a structural scaffold for multiple transcription factors [44]. On the other hand, the MTG16-mediated inhibition of glycolytic gene expression required intact NHR2-3 [1] consistent with that the importance of the latter modules for oligomerization, protein—protein interaction with other co-repressors such as N-CoR and SMRT [15] and interactions with HDACs [15,44,45] required for transcriptional repression. The finding by EMSA that MTG16 associated with HREs within glycolytic gene promoters and the findings by ChIP assays that HIF1α-MTG16 interaction also takes place at these promoters near HRE sites indicates that MTG16 may affect transcriptional activity of HRE-bound HIF1α. Furthermore, recruitment of MTG16 to HREs was suggested to be HIF1α dependent because it was lost at 24 h of incubation with doxycycline at 20% O_2_ (Fig 5) when HIF1α was degraded. HIF1α is normally kept inactive during non-hypoxic conditions by post-translational hydroxylation at conserved critical prolyl residues catalysed in particular by the prolyl—hydroxylase—domain (PHD)–containing enzyme prolyl hydroxylase PHD2 [46]. Hydroxylation is followed by ubiquitination by the von-Hippel—Lindau tumour suppressor protein (VHL) and proteasomal degradation of HIF1α [47]. As PHD proteins require oxygen for catalytic activity, HIF1α is normally stabilized and active when PHD proteins become inactivated during hypoxia [48]. However, in contrast to normal, certain neoplastic cells show O_2_ independent constitutive expression of HIF1α [33]. MTG16-mediated degradation was observed both at 20% O_2_ and during hypoxia. That MTG16 in both cases augmented recruitment of PHD2 to HIF1α and amplified its degradation through a PHD/VHL—dependent pathway is consistent with the combined interaction that we discovered between HIF1α and PHD2. The hydroxylation of Proline-564 of HIF1α by MTG16 observed not only at 20% O_2_ but also at mild hypoxia is consistent with the HIF1α degradation observed. Earlier studies have indicated hydroxylation of HIF1α at Proline-564 during conditions of mild hypoxia [49,50].

Both O_2_/PHD/VHL—dependent and—independent mechanisms regulate HIF1α protein levels. For example, the OS-9 protein is a negative O_2_-dependent regulator of HIF1 that increases prolyl hydroxylation by interacting with HIF1α and PHDs [51]. Hsp70 promotes ubiquitination and proteasomal degradation of HIF1α by recruiting the ubiquitin ligase CHIP [52]. The tumour suppressor Sirtuin 6 acts as a HIF1α co-repressor at glycolytic target genes through deacetylation of Lys9 of Histone 3 [53]. Furthermore, the mitochondrial tumour suppressor Sirtuin 3 promotes destabilization of HIF1α [54,55]. Our results add MTG16 as a novel negative regulator of HIF1α⅑ possibly acting by a PHD/VHL—dependent mechanism. MTG16 is known as a chromatin repressor, which can bind other co-repressors and recruit HDAC [13] with an epigenetic role in gene transcription. Our results indicate an additional function for MTG16 on HIF1-mediated transcription merely by degrading HIF1α. It remains to be investigated whether the MTG isoforms MTGR1 and MTG8 also contribute to HIF1α hydroxylation and proteasomal degradation. Furthermore, it may be of interest to investigate whether the leukemia fusion protein AML1-MTG16 [10] retains the MTG16 ability for degradation of HIF1α.

The present results suggest a mechanism through which MTG16 could contribute to prolonged downregulation of glycolytic genes. This is consonant with MTG16-mediated inhibition of the expression of the glycolytic genes and the PDK1 gene concomitant with a metabolic switch towards mitochondrial respiration [1]. In summary, we identified the co-repressor MTG16 to be a novel binding partner of HIF1α and a negative regulator of its stability. Our results depict molecular mechanisms that have a potential to modulate functional interactions between key proteins regulating the metabolism of neoplastic cells.

## Supporting Information

S1 Table Primers used for ChIP-PCR amplification. Primers were designed using Primer 3 plus software.
(DOCX)

S1 Fig MTG16 inhibition of LDH, HK and PFK expression. The time course is shown for transcriptional expression of *LDH*, *HK* and *PFK* in Raji/MTG16 Tet-On 3G cells during incubation with 20 ng/ml doxycycline under hypoxic conditions (4% O_2_). Doxycycline induction of MTG16 diminished hypoxia induction of *LDH*, *HK* and *PFK* expression. Data are represented as mean ± SEM for n = 3.
(TIFF)
