# Supplementary material for: Myeloid Translocation Gene-16 Co-Repressor Promotes Degradation of Hypoxia-Inducible Factor 1
Source: PLoS One. 2015 May 14;10(5):e0123725. doi: 10.1371/journal.pone.0123725 (PMC4431712; doi:10.1371/journal.pone.0123725)
Supplement: S1 Table — Primers were designed using Primer 3 plus software. (DOCX) [file pone.0123725.s001.docx]

Table S1. List of primers used for real time CHIP-PCR amplification of HRE region of PDK1, PFKFB3, PFKFB4, HK, PFK, LDHA and control regions.

| **Gene Name** | **Accession No.** | **Primer sequences** |
| --- | --- | --- |
| 6-phosphofructo-2-kinase/fructose-2,6-biphosphatase 3  (PFKFB3) | NC_018921.2 | F 5’ GGGTCGGGAGGAGTGGAG 3’  R 5’ CTGAGGCTGGGCTGTGAT 3’ |
| 6-phosphofructo-2-kinase/fructose-2,6-biphosphatase 4  (PFKFB4) | NC_018914.2 | F 5’TCCCTAGCAAGGAGGTAGCA3’  R 5’ CAAACTCAGCTCTCCCAACC 3’ |
| Pyruvate dehydrogenase kinase 1 (PDK1) | NC_18913.2 | F 5’CGCCCTGTCCTTGAGCC3’  R 5’CGGTATGGAGCGTCCCCT3’ |
| Hexokinase 1 (HK1) | NC_18921.2 | F 5’ACTAGCCCTAGGGGCTTCTC3’  R 5’CGCATCATCCTGCACGTC3’ |
| Phosphofructokinase, platelet (PFKP) | NC_18921.2 | F 5’GGGGCTCGTGGAGACGAC3’  R 5’CTTCCCTTCGCCCTTGAG3’ |
| Lactate dehydrogenase A (LDHA) | NC_18922.2 | F 5’AGTCTGCCGGTCGGTTGT3’  R 5’GTGGAACAGCTATGCTGACG3’ |
| Control Primer  Pyruvate kinase Muscle 2 (PKM2) No binding Region | NC_18921.2 | F 5’GGAACTTTCGTGCGTCTTG3’  R 5’GCTCACCTCTCCTGATTACTAC3’ |
