# Supplementary figures and images for: Myeloid Translocation Gene-16 Co-Repressor Promotes Degradation of Hypoxia-Inducible Factor 1
Source: PLoS One. 2015 May 14;10(5):e0123725. doi: 10.1371/journal.pone.0123725 (PMC4431712; doi:10.1371/journal.pone.0123725)

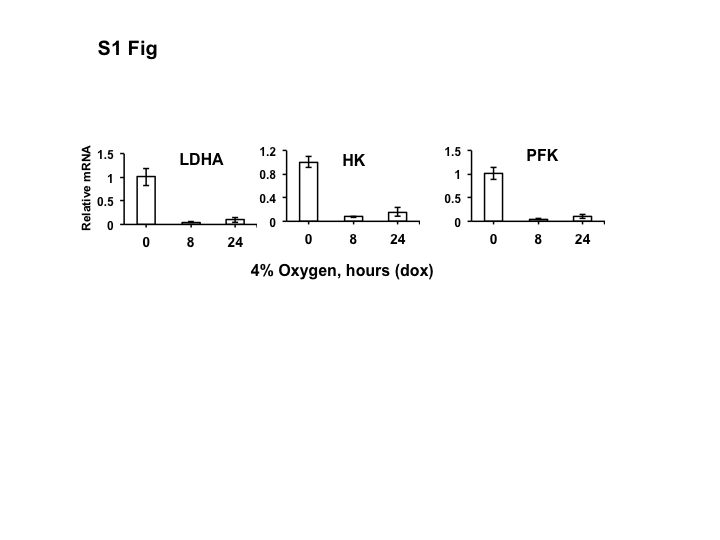

Supplement: S1 Fig — The time course is shown for transcriptional expression of LDH, HK and PFK in Raji/MTG16 Tet-On 3G cells during incubation with 20 ng/ml doxycycline under hypoxic conditions (4% O2). Doxycycline induction of MTG16 diminished hypoxia induction of LDH, HK and PFK expression. Data are represented as mean ± SEM for n = 3. (TIFF) [file pone.0123725.s002.tiff]
